# Expression of EIF5A2 associates with poor survival of nasopharyngeal carcinoma patients treated with induction chemotherapy

**DOI:** 10.1186/s12885-016-2714-2

**Published:** 2016-08-22

**Authors:** Pei-Yu Huang, Ting-Ting Zeng, Xiaojiao Ban, Meng-Qing Li, Bao-Zhu Zhang, Ying-Hui Zhu, Wen-Feng Hua, Hai-Qiang Mai, Li Zhang, Xin-Yuan Guan, Yan Li

**Affiliations:** 1 State Key Laboratory of Oncology in South China, Sun Yat-sen University Cancer Center, Collaborative Innovation Center of Cancer Medicine, Guangzhou, China; 2 Department of Nasopharyngeal Carcinoma, Sun Yat-sen University Cancer Center, Guangzhou, China; 3 Department of Medical Oncology, Sun Yat-sen University Cancer Center, Guangzhou, China; 4 Department of Clinical Oncology, The University of Hong Kong, Hong Kong, China; 5 Room 706, Building 2, No.651 East Dongfeng Road, Guangzhou, 510060 Guangdong China

**Keywords:** Nasopharyngeal carcinoma, Gene amplification, EIF5A2, Prognosis

## Abstract

**Background:**

Nasopharyngeal carcinoma (NPC) is a type of head-neck cancer with a distinguishable geographic and racial distribution worldwide. Increasing evidence supports that the accumulation of additional genetic and epigenetic abnormalities is important in driving the NPC tumorigenic process. In this study, we aim to investigate the association between EIF5A2 (Eukaryotic translation initiation factor 5A2) expression status and NPC clinical outcomes.

**Methods:**

The expression status of EIF5A2 was investigated in the NPC tissue microarray. Tissues were from 166 NPC patients staging II-IV, collected between 1999 and 2005. All patients were administered 2–3 cycles of DDP (cisplatin) + 5-Fu (5-fluorouracil) induction therapy and then treated with a uniform conventional two-dimensional radiotherapy. Cell motility assay, tumor growth assay and cytotoxicity assay were performed on the EIF5A2 overexpressed cells and control cells. siRNA was also used in the in vitro studies.

**Results:**

Positive staining of EIF5A2 was observed in 85.4 % (105/123) informative tumor cases. Multivariate analyses demonstrated that EIF5A2 was an independent prognostic marker of poor overall survival (OS) (*P* = 0.041), failure-free survival (FFS) (*P* = 0.029), and distant failure-free survival (D-FFS) (*P* = 0.043) in patients with locoregionally advanced NPC patients treated with cisplatin + 5-Fu chemoradiotherapy. The forced expression of EIF5A2 in NPC cells enhanced the cells’ motility and growth ability. Knock-down of EIF5A2 in NPC cells decreased the cell’s motility and growth ability. Our results also demonstrated that EIF5A2 overexpression induced chemoresistance of NPC cells to 5-Fu.

**Conclusions:**

Our findings suggested that EIF5A2 expression, as examined by immunohistochemistry, could function as an independent prognostic factor of outcomes in NPC patients with cisplatin + 5-Fu chemoradiotherapy. EIF5A2 might be a novel therapeutic target for the inhibition of NPC progress.

**Electronic supplementary material:**

The online version of this article (doi:10.1186/s12885-016-2714-2) contains supplementary material, which is available to authorized users.

## Background

Nasopharyngeal carcinoma (NPC) is a distinct epithelial malignancy arising in the head and neck region. The disease demonstrates a strong geographic preference, with a high prevalence in Southeast Asia and Southern China with an annual incidence of 20 per 100, 000 people [1, 2]. The mainstay of treatment for NPC is either radiotherapy or combined chemo-radiotherapy, which demonstrates a cure rate of over 90 % in early-stage patients [3]. However, the outcomes for patients with loco-regional advanced disease are still unsatisfactory even with the advanced radiotherapy technique and combined chemotherapy [4, 5]. Significant rates of metastasis or local relapse occur in these patients after radiotherapy or combined chemo-radiotherapy.

Amplification of 3q26 was one of the most frequently detected chromosomal aberration in NPC [6–9], suggesting that candidate oncogene might exist in this region. Eukaryotic translation initiation factor 5A2 (*eIF5A2*) is an oncogene at 3q26. It shares 83 % amino acid identity with EIF5A including the critical functional domain necessary for maturation by hypusine modification [10, 11]. Unlike EIF5A that is universally expressed in tissues, EIF5A2 is only found in testis, brain and tumor tissues [12]. It has been reported to be associated with many cancers: ovarian cancer [13], hepatocellular carcinoma [14], non-small cell lung cancer [15], bladder cancer [16] and colorectal carcinoma [17]. Although 3q26 amplification has been reported in NPC, the expression of *eIF5A2*, located at 3q26.2, has not been investigated in NPC. In the present study, we investigated the expression of EIF5A2 in NPC tissue microarray. Our results indicated that EIF5A2 was an independent adverse prognostic marker of overall survival (OS), failure-free survival (FFS), and distant FFS (D-FFS) in NPC patients. Functional study demonstrated that overexpression of EIF5A2 increased cells’ motility and growth ability. Our results also demonstrated that EIF5A2 overexpression induced chemoresistance to 5-Fu (5-fluorouracil) in NPC cells.

## Methods

### Cell culture

The immortalized nasopharyngeal epithelial cell line NP69 was a gift from Prof. G.S. Tsao [18]. The cells were cultured in keratinocyte serum-free medium supplemented with bovine pituitary extract (Invitrogen, Carlsbad, CA). The NPC cell lines C666, CNE2, and HONE1 were cultured in RPMI-1640 medium (Invitrogen, Carlsbad, CA) with 10 % fetal bovine serum (FBS) (ExCell Bio, China). The cells were cultured at 37 °C in a humidified chamber with 5 % CO2.

### Tissue collection and immunohistochemistry (IHC)

NPC tumor tissues and non-tumor nasopharyngeal epithelial tissues were collected at the Sun Yat-sen University Cancer Center. The Committees for Ethical Review of Research Involving Human Subjects at the Sun Yat-sen University Cancer Center approved the use of the tissue samples in the study. Written informed consent was obtained from patients for the procurement of the tissue samples. A tissue microarray (TMA) was constructed as previously reported [19]. Tissues from 166 NPC patients staging II-IV (AJCC/UICC-5), collected at the time of diagnostic biopsy between 1999 and 2005, were included in the TMA study. All patients were non-metastatic at the time of diagnosis. All patients were administered 2–3 cycles of DDP (cisplatin) + 5-Fu induction therapy and then treated with a uniform conventional two-dimensional radiotherapy protocol [20], in accordance with the treatment policy for NPC at Sun Yat-sen University Cancer Center. The regimen used for induction chemotherapy was PF (DDP 100 mg/m^2^ IV on day 1 and 5-Fu 800 mg/m^2^/d continuously IV on days 1–5). The accumulated radiation doses were 66–78 Gy to the primary tumor and 60–70 Gy to the involved areas of the neck. Conventional fractionated radiotherapy (2 Gy once daily, 5 times per week) was applied in all cases. There were no treatment delays or chemotherapy dose modifications secondary to toxicity in the study. The median follow-up time was 6.6 years.

Slides were deparaffinized in xylene, rehydrated using graded alcohol, immersed in 3 % hydrogen peroxide for 10 min to block endogenous peroxidase activity, and antigen retrieved in EDTA buffer for 15 min. The slides were incubated with 10 % goat serum at RT for 30 min, followed by incubation in diluted EIF5A2 antibody (1:50)(Wolwo, China) overnight at 4 °C. After incubation with horseradish peroxidase-linked secondary antibody (Real EnVision Detection Kit, Gene Tech, China) for 30 min, the slides were counterstained with Mayer’s hematoxylin. The IHC results were evaluated independently by two pathologists. Expression of EIF5A2 was scored as absent (absence of staining), very weak (faint staining in <25 % of tumor cells), moderate (moderate staining in 25 % ~75 %, or strong staining in <25 % of tumor cells), and strong (moderate staining in ≥75 %, or strong staining in ≥25 % of tumor cells). In the present study, moderate/strong staining was defined as positive staining and absent/very weak staining as negative staining [21].

### Western blotting

Cells were lysed in RIPA buffer (Cell Signaling Technology, MA), and the cell lysates were separated by 12 % SDS-PAGE and electrophoretically transferred to PVDF membranes (Bio-Rad, CA) for blotting. Western blot results were captured by traditional exposure or Automatic Chemiluminescence Image Analysis System (Tanon 5200) (Tanon Science & Technology Co., Ltd). EIF5A2 (Wolwo, China) (1:1000) and actin (Cell Signaling Technology, MA) were used in the study.

### NPC cell lines transduced with lentivirus expressing EIF5A2

LV105-*eIF5A2* and LV105 (the empty vector) were bought from GeneCopoeia (Guangzhou, China) and lentivirus was packaged as the manufacturer’s protocol. NPC cells were transduced with lenti-*eIF5A2* and EIF5A2 overexpressed NPC cells were established. The lentivirus packaged with LV105 was used as vector control.

### siRNA transfection assay

siRNA targeting *eIF5A2* was ordered from GenePharma (Shanghai, China). The sequences of siRNA were as follows: 5’ CAUUCAAGAUGGUUACCUUtt 3’ (sense); 5’AAGGUAACCAUCUUGAAUGca 3’ (antisense). The cells were transfected with siRNA using lipofectamine™ 2000 (Invitrogen, Carlsbald, CA) as the protocol supplied by the manufacturer.

### Cell migration assay

Transwell Permeable Support (24-well plate) (Corning Incorporated, NY) was used to assess the rate of cell migration. Cells in serum-free medium were seeded into the upper chamber, while the lower chamber was filled with medium with 10 % FBS. After incubation at 37 °C, penetrated cells to the lower surface of the filter were fixed, stained and counted under a microscope. The assay was repeated three times.

### The anchorage-dependent and -independent growth assays

For the anchorage-dependent growth assay, 1 × 10^3^ cells were seeded into wells of 6-well plate. 10 days later, the surviving colonies were fixed, stained with Crystal violet and counted. Triplicate independent assays were performed. For the anchorage-independent growth assay, 1 × 10^4^ cells were mixed with 0.4 % bactoagar on a bottom layer of solidified 0.6 % bactoagar in 6-well plates. After 2 weeks, colonies consisting of more than 50 cells were counted and the assay was repeated three times.

### Cytotoxicity assay

Cytotoxicity was determined using an XTT assay (CCK-8, Dojindo, Kyushu, Japan). Cells were plated in 96-well plates at the appropriate density. Twenty-four hours later, cells were treated with 2-fold diluted concentrations of 5-Fu (SunRise Ltd., Shanghai, China) for another 48 h at 37 °C. The XTT assay was performed according to the manufacturer’s instructions. Three independent assays were performed.

### Statistical analysis

The data analysis was performed using the SPSS statistical software package (SPSS 16.0, IL). Survival curves were generated according to the Kaplan-Meier method, and the statistical analyses were performed using the log-rank test. Univariate and multivariate survival analyses were performed using Cox proportional hazards regression models. The corresponding hazard ratio (HR) and 95 % CI were generated from the Cox regression models. Pearson Chi-square test was used to analyze the relationship between EIF5A2 expression and clinic-pathological features. Students’ *t*-test was used to analyze data from in vitro assays. A *P* value less than 0.05 was considered statistically significant.

## Results

### The expression of EIF5A2 was up-regulated in NPC cell lines

**Fig. 1:**
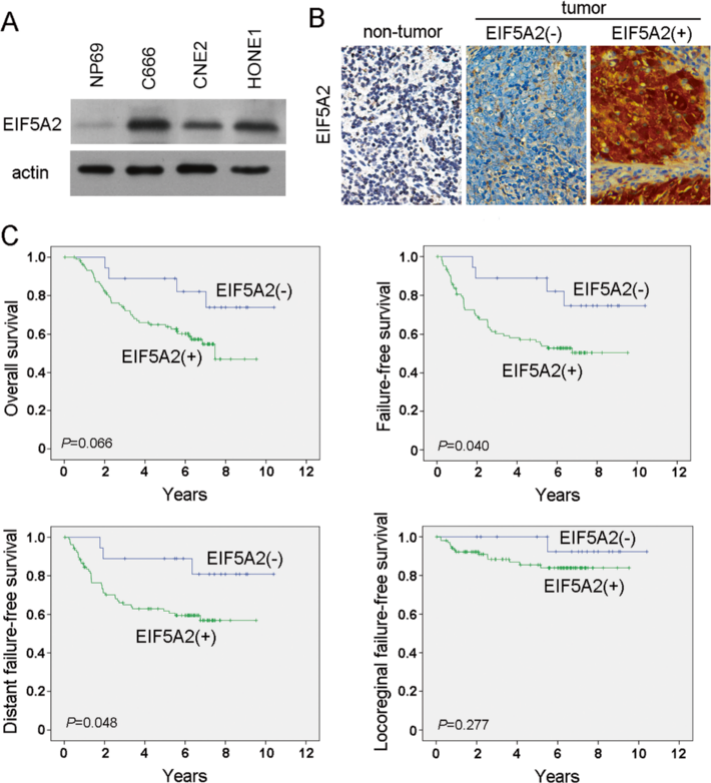
EIF5A2 expression was associated with poorer survival in NPC. **a** The EIF5A2 protein level was determined in immortalized nasopharyngeal epithelial cell line NP69 and NPC cell lines (C666, CNE2 and HONE1). Actin was used as the loading control. **b** Representative pictures of immunostaining of EIF5A2 in non-tumor nasopharyngeal tissue and in NPC tumor tissues (original magnification, 40 × objective). **c** Kaplan-Meier survival analysis of EIF5A2 expression in patients with NPC: EIF5A2 expression and the probability of OS of NPC patients (*P* = 0.066), FFS (*P* = 0.040), D-FFS (*P* = 0.048), and LR-FFS (*P* = 0.277) in NPC patients

Western blotting analysis was used to detect the EIF5A2 expression in NPC cell lines. Compared with the immortalized nasopharyngeal epithelial cell line NP69, the expression of EIF5A2 was increased in the three tested NPC cell lines (C666, CNE2 and HONE1) (Fig. 1a).

### The expression of EIF5A2 was associated with poor response rate and poor survival in NPC

The expression of EIF5A2 was also investigated by IHC using a tissue microarray containing 166 NPC patients. Informative TMA results were obtained in 123 tumor cases. The non-informative samples included lost samples and samples with too few cells or with inappropriate staining. Positive staining for EIF5A2 was observed in 105 of 123 (85.4 %) informative tumor tissues (Fig. 1b).

To examine the clinical significance of EIF5A2 expression in NPC, the correlation of EIF5A2 expression with clinicopathologic features was investigated. The association study showed that EIF5A2 expression was not significantly associated with age, gender, T stage, N stage, staging or WHO classification among the patients (Table 1). Among the 105 patients with EIF5A2 expression, 68 (64.8 %) had an objective response, as compared with 16 of 18 (88.9 %) patients without EIF5A2 expression (*P* = 0.042). The Kaplan-Meier survival analysis revealed that EIF5A2 expression was associated with marginally poorer overall survival (OS) in patients with NPC (log-rank test, *P* = 0.066) (Fig. 1c). EIF5A2 expression was significantly associated with poorer failure-free survival (FFS) (log-rank test, *P* = 0.040) and distant failure-free survival (D-FFS) (log-rank test, *P* = 0.048) (Fig. 1c). Locoregional failure-free survival (LR-FFS) was also analyzed, and no significant difference was observed between groups with positive and negative EIF5A2 staining (log-rank test, *P* = 0.277) (Fig. 1c). Univariate and multivariate analyses (age, gender, T and N tumor categories, and EIF5A2 expression as covariables) were performed, and the results are listed in Tables 2 and 3. We found that EIF5A2 expression was a prognostic factor for poor OS (*P* = 0.041), FFS (*P* = 0.029), and D-FFS (*P* = 0.043), independent of age, gender, and T and N stages, as evidenced by the multivariate analysis (Table 3).

**Table 1:** Association of EIF5A2 expression with clinicopathologic characteristics of NPC patients

		EIF5A2 expression		
Variable	Total cases	EIF5A2 (−)	EIF5A2 (+)	*P* value^a^
Age				0.486
Mean(range)	45.6(21–75)	47 (33–59)	45.3 (21–75)	
Gender				0.692
Male	103	14 (13.6 %)	89 (86.4 %)	
Female	20	4 (20.0 %)	16 (80.0 %)	
T stage				0.616
T1	3	0 (0)	3 (100 %)	
T2	19	4 (21.1 %)	15 (78.9 %)	
T3	59	9 (15.3 %)	50 (84.7 %)	
T4	42	5 (11.9 %)	37 (88.1 %)	
N stage				0.551
N0	10	1 (10.0 %)	9 (90.0 %)	
N1	45	6 (13.3 %)	39 (86.7 %)	
N2	43	5 (11.6 %)	38 (88.4 %)	
N3	25	6 (24.0 %)	19 (76.0 %)	
Staging				0.391
II	2	1 (50.0 %)	1 (50.0 %)	
III	59	7 (11.9 %)	52 (88.1 %)	
IV	62	10 (16.1 %)	52 (83.9 %)	
WHO classification				1.0
II	7	1 (14.3 %)	6 (85.7 %)	
III	116	17 (14.7 %)	99 (85.3 %)	

^a^Chi-square test

**Table 2:** Univariate and multivariate analyses of different prognostic variables on patients’ overall survival (OS)

Variables	Univariate analysis		Multivariate analysis	
	HR (95 % CI)	*P* ^a^	HR (95 % CI)	*P* ^a^
Age	1.028 (1.000–1.056)	0.049	1.042 (1.010–1.075)	0.011
Gender	0.681 (0.348–1.335)	0.263	0.866(0.383–1.957)	0.730
T stage	1.084 (0.789–1.490)	0.619	1.303(0.869–1.954)	0.200
N stage	1.12 (0.835–1.500)	0.450	1.381(0.990–1.927)	0.058
EIF5A2 expression	2.549(0.908–7.158)	0.076	2.949(1.046–8.313)	0.041

^a^Cox regression model; HR, Hazards ratio; CI, confidence interval

**Table 3:** Cox multivariate analyses of EIF5A2 on NPC patients’ survival

	Significant factors	Hazards ratio	95 % CI	*P* ^a^
OS	Age, per year increase	1.042	1.010–1.075	0.011
	EIF5A2(+) vs. EIF5A2(−)	2.949	1.046–8.313	0.041
FFS	EIF5A2(+) vs. EIF5A2(−)	3.128	1.122–8.715	0.029
D-FFS	EIF5A2(+) vs. EIF5A2(−)	3.375	1.040–10.954	0.043
LR-FFS	none			

*OS* overall survival, *FFS* failure free survival, *D-FFS* distant failure-free survival, *LR-FFS* localregional failure-free survival

^a^The covariables: age, gender, T and N categories of NPC tumor, and EIF5A2 expression

### EIF5A2 enhanced NPC cells’ motility

**Fig. 2:**
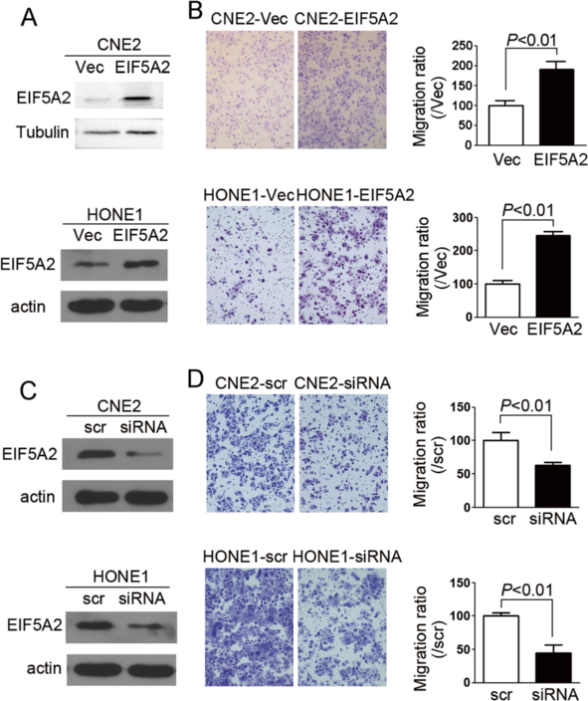
EIF5A2 increased cells’ motility. **a** EIF5A2 protein level was determined in the CNE2 and HONE1 cells transduced with lenti-*eIF5A2* and vector control. Tubulin or actin was used as loading control. **b** Representative pictures and summary of cell migration assay demonstrated that cell motility was increased in EIF5A2 overexpressed cells compared with vector control cells. **c** EIF5A2 was knocked down by siRNA in CNE2 and HONE1 cells. The protein level was determined by WB. Actin was used as loading control. **d** Representative pictures and summary of cell migration assay demonstrated that knock-down of EIF5A2 decreased cells’ motility. Original magnification: 10× objective

Because EIF5A2 was reported to promote cells’ motility in HCC and ESCC [14, 21], we were wondering whether it could affect NPC cells’ motility. Cell migration assay was performed on CNE2 cells transduced with lenti-*eIF5A2* and vector control cells. EIF5A2 protein level was determined by western blotting (Fig. 2a). The results demonstrated that cells migrating through transwell increased in EIF5A2 enforced expression cells, compared with vector control cells (Fig. 2b, *P* < 0.01). Similar results were observed in HONE1-EIF5A2 and HONE1-Vec cells (Fig. 2a and b, *P* < 0.01). We next knocked down EIF5A2 with siRNA and found that cell motility decreased in CNE2-siRNA and HONE1-siRNA cells compared with their corresponding control cells (Fig. 2c and d, *P* < 0.01).

### EIF5A2 promoted tumor cell growth

**Fig. 3:**
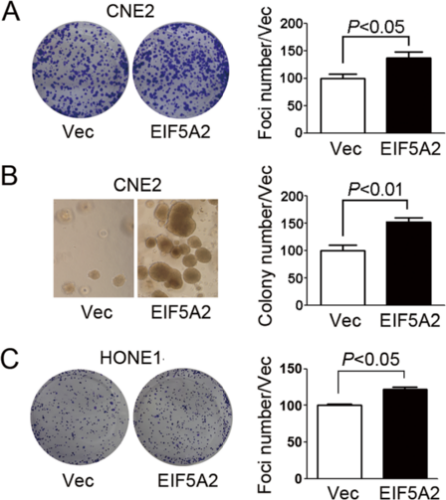
EIF5A2 promoted tumor cell growth. **a** Representative pictures and summary of foci formation assay performed with CNE2-EIF5A2 and vector control cells (CNE2-Vec). **b** Representative pictures and summary of colony formation in soft-agar assay performed with CNE2-EIF5A2 and CNE2-Vec cells. **c** Representative pictures and summary of foci formation assay demonstrated that more foci were formed in HONE1-EIF5A2 than HONE1-Vec cells

**Fig. 4:**
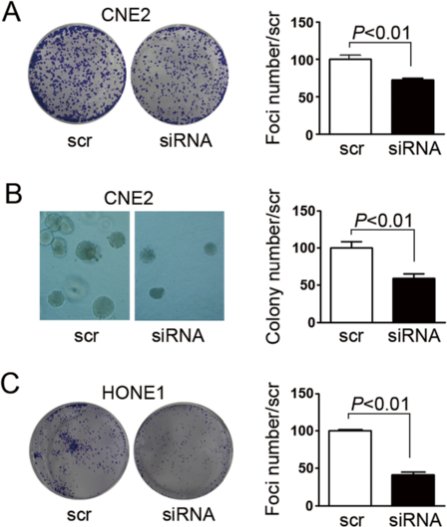
Silencing EIF5A2 decreased tumor cell growth ability. **a** Representative pictures and summary of foci formation assay demonstrated that knock-down EIF5A2 decreased foci numbers in CNE2 cells. **b** Representative pictures and summary of soft agar assay demonstrated that colony number decreased in EIF5A2 knock-down CNE2 cells. **c** Representative pictures and summary of foci formation assay demonstrated that knock-down EIF5A2 decreased foci numbers in HONE1 cells

To determine the effect of EIF5A2 on NPC tumor cell growth, anchorage-dependent and -independent growth assays were performed on EIF5A2 overexpressed CNE2 cells (CNE2-EIF5A2) and vector-alone transfected cells (CNE2-Vec). Results show that number of foci (*P* < 0.05) (Fig. 3a) and colonies formed in the soft-agar (*P* < 0.01) (Fig. 3b) increased in the EIF5A2 overexpressed cells compared with the vector control cells. Taken together, EIF5A2 overexpression increased the growth ability of anchorage-dependent and -independent in CNE2 cells. When we overexpressed EIF5A2 in HONE1 cells, similar foci formation result was observed in HONE1-EIF5A2 cells compared with vector control cells (Fig. 3c, *P* < 0.05). Silencing EIF5A2 in CNE2 also decreased the anchorage-dependent and -independent growth ability of cells (Fig. 4a and b, *P* < 0.01). Foci number decreased in EIF5A2 knock-down HONE1 cells compared with scramble control cells (Fig. 4c, *P* < 0.01).

### EIF5A2 overexpression induced chemoresistance to 5-Fu in NPC cells

**Fig. 5:**
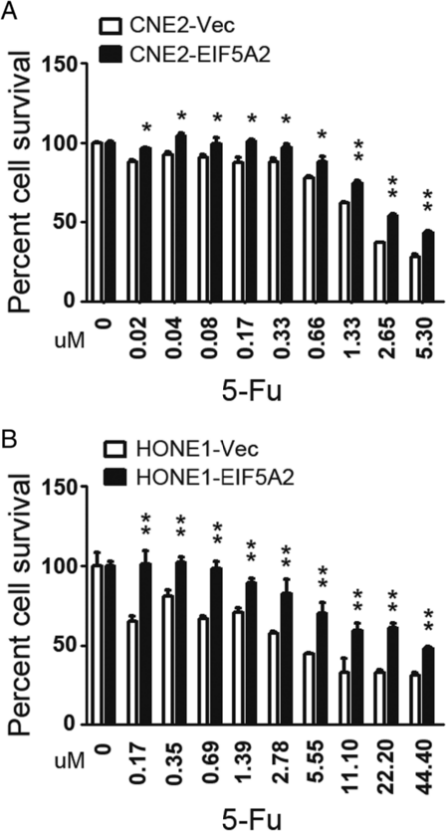
EIF5A2 overexpression induced chemoresistance to 5-Fu in NPC cells. **a** CNE2-EIF5A2 cells are more chemoresistant than CNE2-Vec cells at individual doses of 5-Fu (μM), as shown by XTT assay. **b** HONE1-EIF5A2 cells are more chemoresistant than HONE1-Vec cells at individual doses of 5-Fu (μM), as shown by XTT assay. (*, *P* < 0.05; **, *P* < 0.01)

We next asked whether EIF5A2 expression could induce chemoresistance in NPC cells. XTT assays were used to evaluate the chemosensitivity to 5-Fu between EIF5A2 overexpressed cells (CNE2-EIF5A2 and HONE1-EIF5A2) and vector control cells (CNE2-Vec and HONE1-Vec). As shown in Fig. 5a and b, the CNE2-EIF5A2 and HONE1-EIF5A2 cells were more resistant to 5-Fu than their corresponding vector cells.

## Discussion

NPC is a human malignancy exhibiting highly elevated incidence rates in areas of southern China (including Guangdong and the Hong Kong area). Epidemiologic studies have indicated that environmental factors, including EBV, exposure to nitrosamines in salted and pickled foods and smoke, are associated with NPC initiation and progression [22–24]. However, increasing evidence supports the hypothesis that persistent latent EBV in nasopharyngeal epithelial cells is dependent on the specific existing genetic changes [25, 26]. Like other solid tumors, NPC often demonstrates complex multiple chromosomal aberrations. Accumulation of additional genetic and epigenetic abnormalities is necessary for the tumorigenic process [7]. Array-based comparative genomic hybridization (CGH), fluorescence in situ hybridization (FISH) and chromosome region-specific probes were performed on NPC cell lines, xenograft and primary tumors by different research groups [6–9], and 3q26 amplification was identified as one of the most frequently observed chromosomal aberrations in NPC, indicating there might be candidate oncogenes located in this region.

According to the previous study, *eIF5A2* (located at 3q26.2), has been significantly associated with the advanced stage of ovarian cancer and has been described as an adverse prognostic marker of survival in stage I non-small cell lung cancer patients and localized invasive bladder cancer [13, 15, 16]. It has been also associated with the metastasis of various solid tumors, including colorectal, esophageal and liver cancers [14, 17, 21]. In the current study, the expression of EIF5A2 was identified to be increased in the NPC cell lines compared with immortalized nasopharyngeal epithelial cells. EIF5A2 enforced expression could enhance NPC cells’ motility, anchorage-dependent and -independent growth ability. The prognostic significance of EIF5A2 expression was also evaluated in the NPC TMA. Kaplan–Meier method analysis showed that EIF5A2 expression was associated with marginally OS in patients with NPC (*P* = 0.066), poorer FFS (*P* = 0.040) and D-FFS (*P* = 0.048). Multivariate analyses further demonstrated that EIF5A2 was an independent adverse prognostic marker of OS (*P* = 0.041), FFS (*P* = 0.029), and D-FFS (*P* = 0.043) in NPC patients. EIF5A2 might therefore act as a prognostic biomarker for NPC patients treated with cisplatin + 5-Fu induction chemotherapy. NPC prognosis has been found to be associated with some biomarkers based on individual tumor characteristics: such as Epstein-Barr virus DNA [27], epidermal growth factor receptor [28] and ERCC1 [29]. Whether EIF5A2 can integrate with these markers and TNM stage to further improve prognostic prediction of NPC patients need to be investigated in the future.

Radiation therapy combined with induction chemotherapy is widely used in NPC treatment. The main treatment failure for locoregionally advanced NPC is metastasis followed by locoregional relapse, even with multiple cycles of chemotherapy [30, 31]. In this study, the patients received the conventional two-dimensional radiotherapy with DDP + 5-Fu induction therapy. The NPC patients with EIF5A2 expression had lower objective response compared with patients without EIF5A2 expression (64.8 % vs 88.9 %, *P* = 0.042). It suggested that EIF5A2 expression might be related to chemotherapy or radiotherapy resistance. In fact, a higher expression level of EIF5A2 has been reported to be correlated with decreased doxorubicin sensitivity in breast cancer cell lines [32], and ablation of EIF5A2 enhanced chemosensitivity of HCC cells to 5-Fu [33]. In this study, the results also demonstrated that EIF5A2 overexpression induced chemoresistance to 5-Fu in NPC cells. Taken together, EIF5A2 might be a potential target of therapy in patients with NPC.

## Conclusions

In the study described here, we revealed for the first time that EIF5A2 was an independent adverse prognostic marker of survival in patients with locoregionally advanced NPC treated with cisplatin + 5-Fu chemoradiotherapy. In addition, EIF5A2 transduction into NPC cells enhanced cells’ motility and growth ability. EIF5A2 overexpression also induced chemoresistance to 5-Fu in NPC cells. Our research suggests a novel therapeutic target for the inhibition of NPC tumor progress.

## Additional file

Additional file 1: Figure S1. (A and B) The immunostaining of EIF5A2 in tissue microarray of NPC tumor tissues. A, NPC TMA-1; B, NPC TMA-2. (TIFF 3109 kb)

## Abbreviations

5-Fu: 5-fluorouracil.
CGH: Comparative genomic hybridization
CI: Confidence interval
DDP: Cisplatin
D-FFS: Distant failure-free survival
EBV: Epstein-Barr virus
EIF5A2: Eukaryotic initiation factor 5A2
FFS: Failure free survival
FISH: Fluorescence in situ hybridization
HR: Hazards ratio
IHC: Immunohistochemistry
LR-FFS: Locoregional failure-free survival
NPC: Nasopharyngeal carcinoma
OS: Overall survival
