# Supplementary figures and images for: Expression of EIF5A2 associates with poor survival of nasopharyngeal carcinoma patients treated with induction chemotherapy
Source: BMC Cancer. 2016 Aug 22;16(1):669. doi: 10.1186/s12885-016-2714-2 (PMC4994420; doi:10.1186/s12885-016-2714-2)

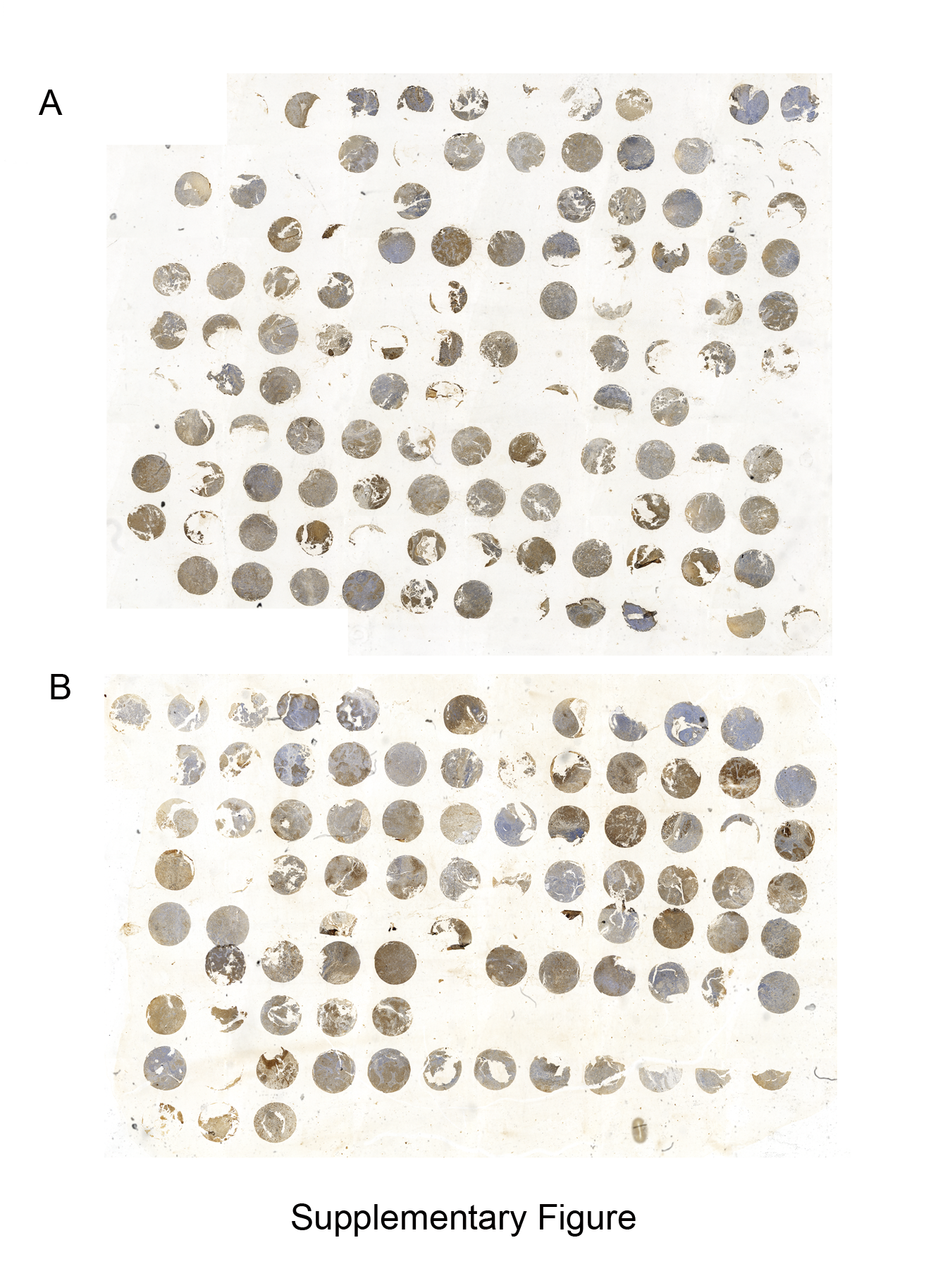

Supplement: Additional file 1: Figure S1. — (A and B) The immunostaining of EIF5A2 in tissue microarray of NPC tumor tissues. A, NPC TMA-1; B, NPC TMA-2. (TIFF 3109 kb) [file 12885_2016_2714_MOESM1_ESM.tiff]
